# Antibacterial and cytotoxic activities of the *Syzygium polyanthum* leaf extract from Malaysia

**DOI:** 10.14202/vetworld.2019.236-242

**Published:** 2019-02-12

**Authors:** Muhammad Luqman Nordin, Abdul Aziz Othman, Arifah Abdul Kadir, Rumaizi Shaari, Abdinasir Yusuf Osman, Maizan Mohamed

**Affiliations:** 1 Department of Clinical, Faculty of Veterinary Medicine, Universiti Malaysia Kelantan, Pengkalan Chepa, 16100 Kota Bharu, Kelantan, Malaysia; 2 Department of Veterinary Preclinical Sciences, Faculty of Veterinary Medicine, Universiti Putra Malaysia, 43400 Serdang, Selangor, Malaysia; 3 Department of Veterinary Paraclinical Science, Faculty of Veterinary Medicine, Universiti Malaysia Kelantan, Pengkalan Chepa, 16100 Kota Bharu, Kelantan, Malaysia

**Keywords:** antibacterial, cytotoxic, mastitis, *Syzygium polyanthum*

## Abstract

**Background and Aim:**

The increasing prevalence of drug resistance eventually leads scientist to discover new drugs that could solve the problem. Since ancient immemorial times, medicinal plants generally known as herbs were widely used in every culture throughout the world. In fact, currently up to 70,000 plant species have been screened for biological activities and about 70% ends up for commercialization. Therefore, this study was aimed to evaluate the potential cytotoxic and antibacterial effect of *Syzygium polyanthum* leaves which are local Malaysia plants, against 4T1 and MCF-7 mammary carcinoma cells, respectively, and also against bacteria causing mastitis in cows.

**Materials and Methods:**

The cytotoxic effect of hydromethanolic extract of *S. polyanthum* against 4T1 and MCF-7 mammary carcinoma cells was evaluated using 3-(4, 5-dimethylthiazol-2-yl)-2,5-diphenyltetrazolium bromide) assay. The cells were treated with the concentration of extracts ranging from 15.63 µg/mL to 1000 µg/ml for 72 h, and the percentage of cell survivability was determined based on minimum concentration that was able to allow at least 50% growth of cancer cells (IC_50_) after 72 h. The antibacterial activity was tested against common bacteria causing mastitis in cow. The bacteria were isolated from milk samples. The antibacterial activity of the extract was determined by disk diffusion method and susceptibility test based on minimum inhibitory concentration (MIC).

**Results:**

*Staphylococcus aureus*, *Staphylococcus hyicus*, and *Staphylococcus intermedius* were isolated from the milk samples that positive for mastitis. The MIC values range from 7.12 mm to 13.5 mm. The extract exhibits the widest zone of inhibition (13.5±0.20 mm) at 1000 mg/ml of concentrations. The extract relatively has low cytotoxicity effect against 4T1 and MCF-7 cells with IC_50_ values ranging from 672.57±59.42 and 126.05±50.89 µg/ml, respectively.

**Conclusion:**

*S. polyanthum* exerts weak antibacterial activity and cytotoxic effect to mammary carcinoma cells. The extract does not toxic to cells. However, further study is recommended, especially, this plant should be tested for *in vivo*.

## Introduction

Mastitis is defined as an inflammation of parenchyma of the mammary glands characterized by physical, chemical, and bacteriological changes in milk and pathological changes in glandular tissues that deteriorate the quality and quantity of milk [1]. Mastitis constantly becomes a serious infectious problem in dairy goats worldwide. The annual economic consequences are approximately $35 billion worldwide which include losses in milk production, sales, and also a high cost for treatments. *Staphylococcus* spp. has been identified to be the main causative agent that contributes to goat’s mastitis. Recently, the abundant uses of antibiotics without control resulting in antimicrobial resistance issue cause failure of mastitis treatment and increase in operation cost, especially, to the farmers due to the absence of effective antimicrobials medication.

Cancer is a non-infectious disease that is very complex and life-threatening to the well-being of the human population. Cancer that occurs in humans and other organisms arises from a single cell which has undergone genetic change due to interaction from external factors and genetic susceptibility of the host. The World Health Organization, 2016 (WHO, 2016), reported that septicemia due to bacterial infections and cancer diseases are the top three most common certified death among human population after cardiovascular disease.

Currently, many anticancer drugs develop drug resistance toward cancer [2,3]. Some of the cancers develop mutation and inherited genetic changes that can modify the drug’s target site. It is also happening to antibiotics. Some of the bacteria develop enzymes that are capable of digesting and destroying antibiotics molecule. Moreover, it is expected to face more resistant bacteria in the near future [4]. Antibacterial resistance is a serious clinical challenge worldwide [5]. Therefore, natural antibacterial products are critical to achieve more diverse antibacterial combinations [5]. Therefore, the emerging of drugs resistance issue has drawn the scientist to find a potential source of treatment to encounter those problems.

A medicinal plant is generally known as a herb. The idea of using herbs as medicines to treat various human ailments including to treat wound, bacterial infections, and cancer diseases is not a new approach. They have been used since ancient civilizations [6]. There are lists of more than 3000 plant species that have reportedly exhibited anticancer properties, and approximately 100 plants with bioactive compounds are on pre-clinical development trials [7,8]. The WHO estimated that 80% of the population, especially, from developing countries rely on traditional medicines mostly plant origin for their health care. Several reports indicating that snake venom contains enzymes and toxins with antimicrobial and anticancer properties and can help to prevent the growth of bacteria have been reported [9,10].

**Figure-1:**
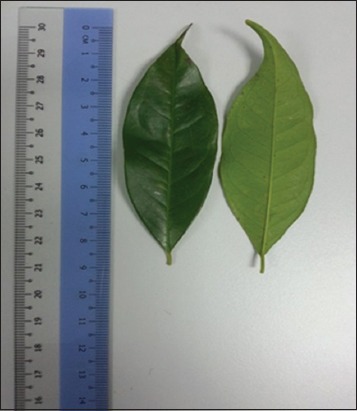
*Syzygium polyanthum* leaves.

*Syzygium polyanthum* (Wight) Walp. var. *Polyanthum* (Figure-1) is wild evergreen shrub which belongs to the family of Myrtaceae. It is well distributed in Indonesia and also Malaysia. It is commonly known as “Daun Salam” or “Indonesian Bay Leaf.” The leaves of “Daun Salam” are often used by Malays as a spice due to its flavor [11,12].

*S. polyanthum* is known traditionally to treat diarrhea, rheumatism, and diabetes [13,14]. The leaves are freshly consumed as “ulam” by traditional Malay people for the treatment of hypertension and general body health maintenance [15]. In Indonesia, local people often add leaves in culinary preparation because they believe that the plant is beneficial in the management of diabetes mellitus, gout, arthritis, and hypertension [16].

This study was conducted with the intention to discover the true potential local herbs for mastitis in dairy cattle, which has antibacterial activity and, at the same time, does not toxic to cells. Perhaps, this study could be further evaluated by the other researchers to find the active compound from these plants. Furthermore, these herbs could be used synergistically with available commercial ones and subsequently improve the prognosis of the treatment and complementary methods such as using herbs or vitamins during treatment is not something new and in many things in help.

This study was performed to provide scientific validation regarding ethnopharmacological values that claim by the traditional practitioner. The findings pertaining to its cytotoxic and antibacterial properties of the *S. polyanthum* leaf extract could provide the true potential of the local herbs. It could be used as alternative medicine or perhaps synergistically used with available treatment to improve the prognosis of the disease.

## Materials and Methods

### Ethical approval

Not applicable in this study.

### Collection and identification

*S. polyanthum* Lam. fresh leaves were collected from Biodiversity Unit, Universiti Putra Malaysia (UPM). The leaves were certified with a deposited voucher specimen (SK 2835/15) from the Herbarium of Natural Products, IBS, UPM. The collected leaves were rinsed with distilled water, cleaned, and then dried in oven for 7 days.

### Extraction of plant leaves

The dried leaves of *S. polyanthum* were pulverized using a commercial blender. About 400 g of pulverized leaves were soaked in methanol:distilled water (80:20, v/v) in a conical flask for 72 h. The flask was continually shaking daily for 3 consecutive days. The solution was filtered using Whatman No. 42 filter paper to separate solvent-containing extract. The extract was evaporated using a vacuum rotary evaporator (Heidolph German) and controlled heating bath at 30°C. The extract yield was stored in the refrigerator until used for the analysis.

### Bacterial strains

The antibacterial potency of plant extract was evaluated using three bacterial strains causing mastitis in cow. The bacterial strains were isolated from milk samples from 10 dairy cows having mastitis. The mastitis cow was detected based on gross signs of udder infection during physical examination, appearance of abnormal milk production, and also California Mastitis Test (CMT). Milk samples were inoculated onto blood agar plates and MacConkey agar, respectively. Inoculated plates were then incubated aerobically at 37°C for 24-48 h. Secondary culture was performed to obtain a pure culture. The purified bacterial strains were confirmed based on colony morphology, gram staining, and biochemical test.

### Antibacterial activity of extracts

The antibacterial assay of hydromethanolic extract was performed according to the method described by Bauer *et al*. [17] with a slight modification. The Mueller-Hinton Agar media, along with the inoculum (106 CFU/ml), were poured into the Petri dishes. For the agar disk diffusion method, sterile filter paper disk was saturated with 125, 250, 500, and 1000 mg/ml of the extract, allowed to dry, and then placed on the upper layer of the seeded agar plate. The plates were incubated overnight at 37°C. Antibacterial activity was determined by measuring the diameter of the zone of inhibition (mm) surrounding bacterial growth strains. The bacterial strains were isolated from milk samples of mastitis dairy cattle.

### Minimal inhibitory concentration (MIC)

*S. polyanthum* extract was tested for the MIC test using the broth dilution method according to Jorgensen and Turnidge [18]. A different concentration of the tested material was obtained by four rows in each containing 125, 250, 500, and 1000 mg/ml. Then, 0.5 ml of bacterial suspension was filled to each tube to achieve a final concentration of 1-5 × 10^5^ CFU/ml. Two sets of controls were set for each tube which contained (a) positive control consisting of broth and bacterial suspension and (b) negative control only consisting of broth. Afterward, the tubes were incubated for 24 h incubation at 37°C. The tubes were observed for visible bacterial growth as evidenced by turbidity. Color changes were observed, and the tubes with colorless appearance were taken as a positive. The lowest concentration of extracts which tubes with colorless indication was recorded as the MIC value. The average values were calculated for the MIC of the test material.

### Minimum bactericidal concentration (MBC)

After identification of the MIC, inoculum from each tube was streaked into agar plate and incubated at 37°C for 24 h. The streaks from each tube that exhibits prevention of bacterial growth was recorded as MBC values. Streaks were taken from the two lowest concentrations of the plant extract plates exhibiting invisible growth. One streak from each tube that exhibits prevention of bacterial growth was recorded as MBC values.

### Cytotoxicity activity

Cytotoxic activity of *S. polyanthum* extract was conducted according to the method described by Baharum *et al*. [19] and Nordin *et al*. [20]. Cytotoxicity activity of *S. polyanthum* extract was prepared with concentrations ranging from 15.63 µg/mL to 1000 µg/mL. The seeding cell density was 1×10^5^ cells/mL of mouse mammary carcinoma cell line (4T1). The cells were obtained from the American Type Culture Collection (ATCC, USA). The cancer cell was grown in RPMI 1640 medium supplemented with L-glutamine, 10% fetal bovine serum, and 1% antibiotic as a complete growth medium. The experiment was repeated thrice, and the percentage of cell survivability versus concentration was calculated according to the following equation [19]:


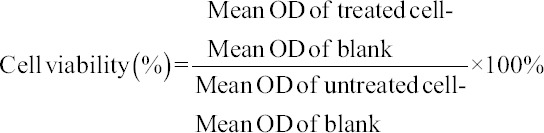


The cytotoxic effect of *S. polyanthum* extract against 4T1 was recorded as IC_50_ and compared with untreated cells according to the method described by Nordin *et al*. [20] and Ayob *et al*. [21].

### Statistical analysis

All the percentages of the zone of inhibition values were expressed as mean (n=3) per plate ± standard deviation (SD) and were analyzed using one-way ANOVA. The test was considered statistically significant when p<0.05 while all the percentages of cell survivability were expressed as mean (n=3) per plate±SD for triplicate and analyzed using one-way ANOVA followed by Dunnett’s multiple comparison test. *p<0.05, **p<0.01, and ***p<0.001 denote significant difference as compared to untreated cell (0 µg/ml).

## Results

### Bacterial culture

Eighteen dairy cows with 68 milk samples were successfully examined, and had clinical mastitis. The diagnosis was further conducted using CMT to confirm the mastitis. The samples were scored as in Table-1. The prevalence of CMT positive was 42.64% and cultured on blood agar. Twenty of 29 samples cultured successfully grown.

**Table-1:** Milk scores using CMT.

Number of animals	Number of milk samples	CMT scores				Positive samples (%)

		0	1	2	3	
18	68	16	23	22	7	42.64

CMT=California mastitis test

### Bacterial identification

On plate morphology, the results showed the presence of *Staphylococcus aureus. S. aureus* was in size about 2 mm, having a circular shape with a flat surface and the color was whitish opaque gray with smooth edge and shiny appearance. *Staphylococcus hyicus* about having the same characteristic with *S. aureus* without giving any hemolysis character on blood agar.

On the other hand, *Staphylococcus intermedius* also seems to be opaque white, low convex and have smooth edges. The colony size is about 2-4 mm in diameter and sometimes bigger than *S. aureus*. This was followed by gram staining which yielded 15 Gram-positive cocci bacteria with catalase positive, 2 Gram-positive cocci with catalase negative, 1 Gram-negative bacteria with small rod, and 2 of them were Gram-positive filamentous bacteria with slow catalase-positive reaction. Various biochemical tests were used to identify the isolates. It started from gram-staining where if the results were positive, then catalase will proceed, and if it positive too, the result of the coagulase test will determine the biochemical test used. Table-2 shows the list of tests used for coagulase-positive bacterial strains.

**Table-2:** Biochemical characteristic of *Staphylococcus aureus*, *Staphylococcus hyicus*, and *Staphylococcus intermedius*.

Cat	Coa	BB	VP	Mal	Man	ADH	Bacteria identification
+						+	*Staphylococcus hyicus*
+	+	+	+	+	+		*Staphylococcus aureus*
+	+	+	+	+	+		*Staphylococcus intermedius*.

Cat=Catalase, Coa=Coagulase, BB=Blood broth, VP=Voges–Proskauer, Mal=maltose, Man=Mannitol, ADH=Arginine Dihydrolase

From these various biochemical tests that have been used to identify the species isolates, it was found that the *S. aureus*, *S. hyicus*, and *S. intermedius* were identified as in Table-2. *S. aureus* was the most common bacteria isolated (6 of 14 isolates) followed by *S. hyicus* and *S. intermedius*, in which both of them were 4 isolates of 14 (Table-3).

**Table-3:** Frequency of bacteria strains isolated from grown CMT-positive milk.

Isolates	Number of isolates (%)
*Staphylococcus aureus*	6 (42.8)
*Staphylococcus hyicus*	4 (28.6)
*Staphylococcus intermedius*	4 (28.6)
Total	14 (100)

### Minimum inhibitory concentration (MIC)

**Figure-2:**
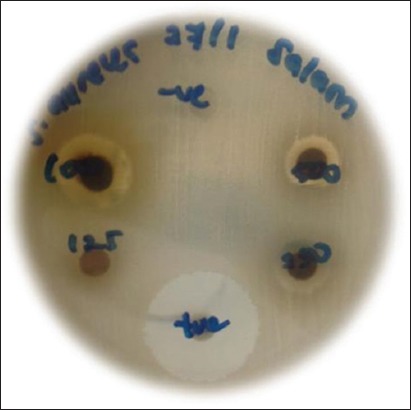
Growth zone of inhibition of some of the bacteria (*Staphylococcus aureus*) caused by *Syzygium polyanthum* extract.

Three most common bacteria isolates which were *S. aureus*, *S. hyicus* and *S. intermedius* were chosen to be tested with hydromethanolic extract of *S. polyanthum* (Table-3). Table-4 shows the mean diameter of the zone of inhibition produced by different concentrations of extract. The zone of inhibition was measured in millimeters (mm) (Figure-2).

**Table-4:** Mean diameter zone of inhibition produced by hydromethanolic extract of *Syzygium polyanthum* against respective bacteria.

Plant extract	Concentration		Zone of inhibition (mm)		
	
	%	mg/ml	*Staphylococcus aureus*	*Staphylococcus hyicus*	*Staphylococcus intermedius*
*Syzygium polyanthum*	100	1000	13.50±0.20^a^	12.00±0.35^a^	12.87±0.31^a^
	50	500	11.62±0.23^ab^	10.12±0.12^a^	10.00±0.40^ab^
	25	250	10.25±0.32^b^	8.25±0.25^b^	8.87±0.23^b^
	12.5	125	8.00±0.61^c, d^	7.62±0.12^b^	7.12±0.29^b^
Amoxicillin	100	10	21.81±0.35^a^	33.87±0.39^b^	18.43±0.31^c^

Value represent mean±SEM (n=4). Means with different alphabets indicate significant differences among different concentrations

The *S. polyanthum* extract shows the largest zone of inhibition at 1000 mg/ml concentration against *S. aureus* which was 13.5 mm in diameter. The sensitivity reduced as the concentration of plant extract decreased. The treatment effect was time-dependent manner. When compared to the positive control, *S. hyicus* was the most sensitive toward amoxicillin followed by *S. aureus* and *S. intermedius*. On the other hand, 3% DMSO which was used as negative control also failed to inhibit any bacteria.

MIC was determined on 96 plates by quantitative evaluation. Table-5 shows that MIC for *S. polyanthum* extract was at a concentration of 125 mg/ml against all bacteria tested. In meanwhile, the MBC was similar which is at 125 mg/ml. The inhibitory concentration of *S. polyanthum* extract started at 125 mg/ml with the zone of inhibition of 8.00±0.61 mm for *S. aureus*, 7.62±0.12 mm for *S. hyicus*, and 7.12±0.29 mm for *S. intermedius*. The MBC of the plant extract was confirmed by the absence of bacterial growth of the isolated bacteria when streaked from inhibition zone corresponding to their lowest MIC values. The MBC of the *S. polyanthum* was also at 125 mg/ml suggested that the plant can be used to prevent and control mastitis in dairy cattle.

**Table-5:** MIC and MBC of plant extract against *Staphylococcus aureus*, *Staphylococcus hyicus,* and *Staphylococcus intermedius*.

Plant extract	MIC (mg/ml) *Syzygium polyanthum*	MBC (mg/ml) *Syzygium polyanthum*
*Staphylococcus aureus*	125	125
*Staphylococcus hyicus*	125	125
*Staphylococcus intermedius*	125	125

MIC=Minimum inhibitory concentration, MBC=Minimum bactericidal concentration

### Effect of crude hydromethanolic extract of S. polyanthum

**Figure-3:**
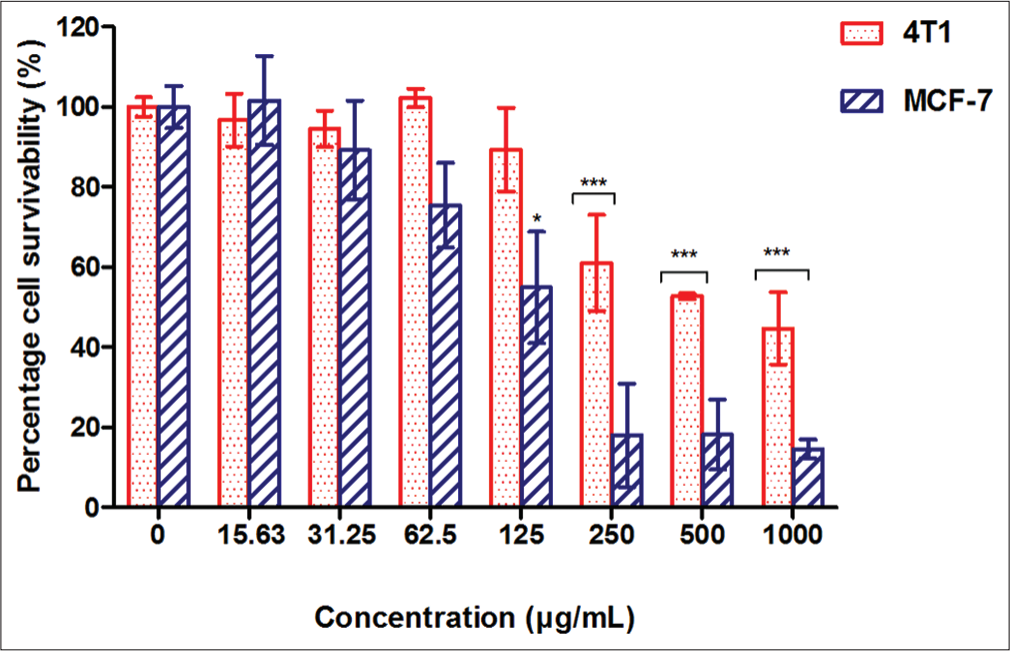
Cell survivability (%) of 4T1 and MCF-7 cancer cells following treatment with *Syzygium polyanthum* extract for 72 h.

**Figure-4:**
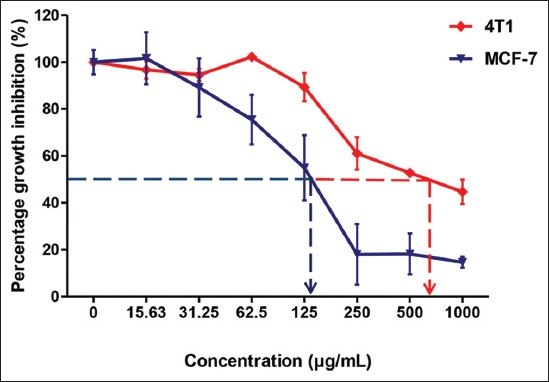
Minimum growth inhibitory concentration (IC_50_) of *Syzygium polyanthum* extract on 4T1 and MCF-7 mammary carcinoma cell lines.

For the cytotoxic screening, *S. polyanthum* extract shows the cell survivability (%) of 4T1 and MCF-7 with a dose-dependent manner (1000-15.63 µg/ml). Overall, the extract does not exhibit strong cytotoxic activities, showing that more than 50% cells were viable when treated with plant extracts at a concentration of 100 µg/ml and above. The IC_50_ values for the extract against both cancer cells were more than 100 µg/ml (Figures-3 and 4). Table-6 shows the IC_50_ values of *S. polyanthum* on 4T1 and MCF-7 mammary carcinoma cell lines. The inhibition of cell viability was more than 50% in 4T1 and MCF-7 after treatment with *S. polyanthum* extract at the concentration 672.57±59.42 µg/mL and 126.05±50.89 µg/mL, respectively.

**Table-6:** IC_50_ values (μg/mL) of *Syzygium polyanthum* extract on 4T1 and MCF-7 mammary carcinoma cell lines.

Cancer cell lines	IC_50_ values of extracts/drug (µg/mL)

	Hydromethanolic extract of *Syzygium polyanthum*
4T1	672.57±59.42
MCF-7	126.05±50.89

All values are expressed as mean (n=3)±SD of triplicate experiments

## Discussion

Results obtained in the present study revealed that the hydromethanolic extract of *S. polyanthum* exhibits antibacterial activity against *S. aureus*, *S. hyicus*, and *S. intermedius*(Table-2). The plant extract was tested at a concentration of 125-1000 mg/ml to evaluate the inhibitory effects against bovine mastitis isolated pathogens. In this study, the MIC of *S. polyanthum* against mastitis bacteria in bovine was 125 mg/ml. The antibacterial activity is considered weak (zone of inhibition between <8 mm) against *S. aureus*. This is in agreement with study by Mehta *et al*. [22] on *Nelumbo nucifera* flowers in which zone of inhibition between 10 and 16 mm is considered moderately potent. Another study conducted from *Adina cordifolia*, *Asparagus racemosus*, *Aegle marmelos*, *Cassia tora*, and *Dillenia pentagyna* extracts which were considered showed weak antibacterial activity in which zone of inhibition was between 5 mm and 8 mm [23]. This means that, in general, plant extract with zone of inhibition between 10 and 16 mm is considered moderately active while plant extract with zone of inhibition between 5 mm and 8 mm is considerate weakly active.

Ramli *et al*. [24] reported that the inhibition zone of *S. polyanthum* extract against *S. aureus* was 9.33±0.52 mm. In this experiment, findings showed that the inhibition zone is proportional with concentration. As the concentration of extract increases, the zone of inhibition is also wider. However, there is no difference in terms of pathogens susceptibility because the MIC and MBC values are the same which are at 125 mg/ml. It means that these bacteria inhibited and killed at the same concentration of extract. This is in agreement with previous study using the same plant extract and Gram distinction between bacteria has an effect on susceptibility rate [24]. The similarity of MIC and MBC could be due to the pathogens tested in this experiment which were Gram-positive bacteria.

In general, *S. polyanthum* extract exhibits cytotoxic activity (when IC_50_ <1000 µg/ml), but according to the United States National Cancer Institute plant screening program and report [25], the *S. polyanthum* extract was in category of weakly active against cancer cells (IC_50_ >100-1000 µg/ml). In this study, the minimum concentration of *S. polyanthum* extract that gives at least 50% of cell inhibition of 4T1 and MCF-7 cells was 672.57±59.42 and 126.05±50.89 µg/ml, respectively. 3-(4,5-dimethylthiazol-2-yl)-2,5-diphenyltetrazolium bromide) (MTT) cell-based colorimetric assay was used in the cytotoxicity assessment. It detects the viable cells based on tetrazolium compound reduction after reacting with metabolized cells. If the cell metabolically actives, it has the ability to convert yellow MTT reagent into purple formazan by mitochondrial succinate dehydrogenase reaction. The intensity of purple formazan color solution was depending on the amount of viable cells that able to actively metabolize the tetrazolium compound in MTT reagent. Finally, high or low number of live cell was measured under spectrophotometer which gave the absorbance value of each well.

Mixture of methanol and water (hydromethanol) was used as solvent for extraction in this study. This means that polar solvent was used since methanol and water were classified as polar solvent. Hydromethanolic extract (mixture of methanol and water) has greater efficiency in pulling out (extract) phytochemical compound in plant compared to pure solvent (without mixture) [20,26,27]. Interestingly, in this study, it was found that both antibacterial and cytotoxic activities of this plant are considered weak. Even though *S. polyanthum* extracts do not demonstrate potent antibacterial and cytotoxic activities, it does not mean that the extract has poor therapeutic value. Perhaps, it is a possibility of phytochemical compound existing in the plants which are multicomponent mixture with different polarities. Selection of an appropriate solvent for extraction is crucially important since phytochemical compounds contribute to the therapeutic value of the plant. Therefore, further phytochemical analyses of this plant need to be conducted to determine phytochemical constituents and further isolation of the bioactive compound is strongly recommended.

## Conclusion

Crude hydromethanolic extract of *S. polyanthum* exerts weak antibacterial activity. Overall, the extract can be considered weak cytotoxic to mammary carcinoma cells. However, it was more toxic to MCF-7 cells compared to 4T1. These results support the claims from traditional practitioner. However, further study is recommended, especially, this plant should be tested for *in vivo*. This is because many studies found that the effect would be different when tested between *in vitro* and *in vivo*, and perhaps, it would be more potent. This is because animals have an immune system and microenvironment such as hormone, and thus, the synergistic effect derived from extract might exist *in vivo* compared to *in vitro*.

## Authors’ Contributions

MLN involved in all aspects of the study including concept, design, data collection, interpretation of data, statistical analysis, and manuscript preparation. AAO and AAK involved in obtaining funding and assisting the experiment. RS, AYO, and MM contributed to the statistical analysis and evaluated the manuscript. All authors have read and approved the final manuscript.

## Data Availability

The datasets generated and/or analyzed during the current study are not publicly available due to its part of a big study (data) but are available from the corresponding author on reasonable request.

## References

[1] Radostits O.M, Arundel J.H (2000). Veterinary Medicine:A Textbook of the Diseases of Cattle, Sheep, Pigs, Goats and Horses.

[2] Zahreddine H, Borden K.L (2013). Mechanisms and insights into drug resistance in cancer. Front Pharm.

[3] Townsend D.M, Tew K.D (2003). The role of glutathione-S-transferase in anticancer drug resistance. Oncogene.

[4] Fathi B, Jamshidi A, Zolfagharian H, Zare Mirakabbadi A (2011). Investigation of the antibacterial effect of venom of the Iranian snake *Echis carinatus*. Iran. J. Vet. Sci. Technol.

[5] Ang J.Y, Ezike E, Asmar B.I (2004). Antibacterial resistance symposium series society for applied microbiology. Ser. Soc. Appl. Microbiol.

[6] Balunas M.J, Kinghorn A.D (2005). Drug discovery from medicinal plants. Life Sci.

[7] Jain R, Jain S.K (2010). Traditional medicinal plants as anticancer agents from Chhattisgarh, India:An overview. Int. J. Phytomed.

[8] Harvey A.L (2008). Natural products in drug discovery. Drug Discov. Today.

[9] Park M.H, Choi M.S, Kwak D.H, Oh K.W, Yoon D.Y, Han S.B, Song H.S, Song M.J, Hong J.T (2011). Anti-cancer effect of bee venom in prostate cancer cells through activation of caspase pathway via inactivation of NF-κB. Prostate.

[10] Ahmed U, Malik Mujaddad-ur-Rehman N.K, Fawad S.A, Fatima A (2012). Antibacterial activity of the venom of *Heterometrus xanthopus*. Indian J. Pharm.

[11] Noorma W.H, Lemmens R.H, Soerianegara M.J, Wong W.C.I (1995). *Synzgium gaertner*;plant resource of South East Asia No 5(2). Timber Trees:Minor Commercial Timbers.

[12] Ismail M (2007). Ensiklopedia Herba:Kegunaan Dan Khasiat Perubatan Tradisi. Anzagain Sdn Bhd.

[13] Dalimartha S (2007). Atlas of Indonesian Medicinal Plants.

[14] Haque M.M (2004). Inventory and documentation of medicinal plants in Bangladesh. Medicinal Plants Research in Asia.

[15] Ismail A, Mohamed M, Sulaiman S.A, Wan Ahmad WAN (2013). Autonomic nervous system mediates the hypotensive effects of aqueous and residual methanolic extracts of *Syzygium polyanthum*(Wight) Walp. var *Polyanthum* leaves in anesthetized rats. Evid. Based Complement. Altern. Med.

[16] Widyawati T, Yusoff N.A, Asmawi M.Z, Ahmad M (2015). Antihyperglycemic effect of methanol extract of *Syzygium polyanthum*(Wight.) leaf in streptozotocin-induced diabetic rats. Nutrients.

[17] Bauer A.W, Kirby W.M.M, Sherris J.C, Turck M (1966). Antibiotic susceptibility testing by a standardized single disk method. Am. J. Clin. Pathol.

[18] Jorgensen J.H, Turnidge J.D, Murray P.R, Baron E.J, Jorgensen J.H, Landry M.L, Pfaller M.A (2007). Antibacterial susceptibility tests:Dilution and disk diffusion methods. Manual of Clinical Microbiology.

[19] Baharum Z, Akim A.M, Taufiq-Yap Y.H, Hamid R.A, Kasran R (2014). *In vitro* antioxidant and antiproliferative activities of methanolic plant part extracts of *Theobroma cacao*. Molecules.

[20] Nordin M.L, Kadir A.A, Zakaria Z.A, Abdullah R, Abdullah M.N.H (2018). *In vitro* investigation of cytotoxic and antioxidative activities of *Ardisia crispa* against breast cancer cell lines, MCF-7 and MDA-MB-231. BMC Complement. Altern. Med.

[21] Ayob Z, Mohd Bohari S.P, Abd Samad A, Jamil S (2014). Cytotoxic activities against breast cancer cells of local *Justicia gendarussa* crude extracts. Evid. Based Complement. Altern. Med.

[22] Mehta N.R, Patel E.P, Patani P.V, Shah B (2013). *Nelumbo nucifera*(Lotus):A review on ethnobotany, phytochemistry and pharmacology. Indian J. Pharm. Biol. Res.

[23] Vashist H, Jindal A (2012). Antimicrobial activities of medicinal plants –Review. Int. J. Res. Pharm. Biomed. Sci.

[24] Ramli S, Radu S, Shaari K, Rukayadi Y (2017). Antibacterial activity of ethanolic extract of *Syzygium polyanthum* L. (Salam) leaves against foodborne pathogens and application as food sanitizer. Bio. Med. Res. Int.

[25] Atjanasuppat K, Wongkham W, Meepowpan P, Kittakoop P, Sobhon P, Bartlett A, Whitfield P.J (2009). *In vitro* screening for anthelmintic and antitumor activity of ethnomedicinal plants from Thailand. J. Ethnopharmacol.

[26] Aktumsek A, Zengin G, Guler G.O, Cakmak Y.S, Duran A (2013). Antioxidant potentials and anticholinesterase activities of methanolic and aqueous extracts of three endemic *Centaurea* L. species. Food Chem. Toxicol.

[27] Al-Barazanjy R.K, Dizaye K, Al-Asadye A (2013). Cytotoxic and cytogenetic effects of *Salvia officinalis* on different tumor cell lines. Middle East J. Int. Med.

